# Update on protracted bacterial bronchitis in children

**DOI:** 10.1186/s13052-020-0802-z

**Published:** 2020-03-30

**Authors:** Xiao-bo Zhang, Xiao Wu, Guang-min Nong

**Affiliations:** Pediatric Department, First Affiliated Hospital of Guangxi Medical University, Nanning, 530021 Guangxi China

**Keywords:** Protracted bacterial bronchitis, Pathogen, Treatment, Children

## Abstract

**Background:**

Chronic cough is a common symptom in children and protracted bacterial bronchitis (PBB) is one of the causes of chronic cough. However, the understanding of this disease remains limited. The present study aims to update PBB in children.

**Methods:**

The clinical data of children with PBB from 2014 to 2018 were retrospectively analyzed, and PBB clinical features of published studies were summarized. Electronic databases were searched in May 2019. Clinical studies were included in the present study. Reviews were undertaken in duplicate.

**Results:**

Totally 712 cases were analyzed in this study, including 52 cases in our center and 660 cases from 14 studies. In the 52 cases, 88.5% of patients with PBB were less than 6 years old and all of them complained of wet cough. Three cases were confirmed with laryngomalacia, and microbiologically-based-PBB were identified in 13 cases (9 *Streptococcus pneumonia*, 3 *Staphylococcus aureus*, and 1 *Pseudomonas aeruginosa*). Twenty cases were completely remitted after treatment. In the 14 studies, the patients with PBB were typically younger than 3 years old, accompanying wheezing and airway malacia. Co-infection was common in most western cases, *Streptococcus pneumonia*, *Haemophilus influenza* and *Moraxella catarrhalis* were the top three pathogens. Symptoms were improved in most patients, whereas some cases with comorbidities required prolonged antibiotics treatment.

**Conclusions:**

PBB is common in male infants with chronic wet cough and accompanied by wheezing and airway deformities. Most cases are clinically diagnosed PBB in China and microbiologically-based-PBB is common in western countries. Co-infection could be found, *Streptococcus pneumoniae* and *Haemophilus influenza* were the most frequent etiology in China and western countries, respectively. Patients with comorbidities may need extended antibiotics treatment for more than 2 weeks.

## Introduction

Protracted bacterial bronchitis (PBB) is an old diagnosis and is considered to be the main cause of chronic wet cough in children [1, 2]. The continual cough of PBB may affect exercise tolerance, disturb sleep, and cause significant levels of morbidity, however, the understanding of the disease remains limited [3, 4]. The lack of awareness in diagnosis and the confusion in antibiotics course for treatment still exist. In order to update on the management of PBB, this study retrospectively analyzed the clinical data of PBB diagnosed in our center, as well as summarized the PBB clinical features of published studies.

## Methods

### Patients

Patients diagnosed with PBB and underwent follow-up (aged <14 years) at the First Affiliated Hospital of Guangxi Medical University between January 2014 and December 2018 were enrolled in this study.

### Inclusion and exclusion criteria

Diagnostic criteria for PPB [5]: (1) clinically-based-PBB: chronic wet cough > 4 weeks; without pointers caused by specific disease; cough improved after 2 weeks of treatment with antibiotics. (2) microbiologically-based-PBB: chronic wet cough > 4 weeks with evidence of lower respiratory tract infection, positive bacterial culture (≥10^4^cfu/ml) in sputum or bronchoalveolar lavage fluid (BALF); cough significantly improved after 2 weeks of antibiotic treatment. (3) Exclusion criteria: patients with mycoplasma infection, infiltrate on chest X-ray or CT, immunodeficiency, congenital heart disease, and other basic diseases.

### Clinical data collection

Demographic characteristics (gender, age of onset, premature birth or not), clinical manifestations (phase and duration of wet or dry cough, wheezing or not, accompanying symptoms, etc.), physical examination findings, auxiliary examination (blood routine examination, CRP, immune function, bronchoscopy, cell classification of alveolar lavage fluid, etiology, chest X-ray or computed tomography), treatment history, and follow-up information were collected for all enrolled patients. Follow-up information included response to antibiotics, duration of cough, complications, and prognosis which were collected by telephone interview or outpatient consulting every 2 weeks, for a total of 4 weeks.

### Information sources of review

We completed a systematic search of databases (CNKI, Wan Fang, Medline, Embase, and Pubmed) in May 2019. Clinical studies with detail data (including diagnostic criteria, demographic characteristics, clinical features, auxiliary examination, treatment, and follow-up information) were included in the present study. Reviews were undertaken in duplicate. We summarized the main observation indexes, including gender, age, course of the disease, pathogenic bacteria, complications, therapeutic drugs, course of antibiotics, and treatment effect.

### Statistical analysis

Descriptive analysis was used for this study. Categorical data were expressed as numbers (percentages).

## Results

### Demographic data of patients with PBB

A total of 52 children of PBB were enrolled in our center, 33 males and 19 females. Fifteen patients were <1 year, 16 patients were between 1 and 3 years of age, 15 patients were 3–6 years old, 6 patients were 6–14 years old. Most of the patients were younger than 6 years old (88.5%) (Table 1).

**Table 1:** Clinical characteristics of PBB in the 52 Chinese patients

Characteristics	PBB
**Gender, M(F)**	33 (19)
**Age**	
<1y	15 (28.8%)
1-3y	16 (30.8%)
3-6y	15 (28.8%)
6-14y	6 (11.5%)
**Course**	
>4 week	19 (36.5%)
4-12 week	14 (26.9%)
>12 week	19 (36.5%)
**Symptoms**	
Wet cough	52 (100%)
Purulent sputum	13 (25%)
Cough both day and night	22 (42.3%)
Wheezing	15 (28.8%)
Fever	11 (21.2%)
Nasal symptoms	19 (36.5%)
**Physical examination**	
Lung moist rales	30 (57.7%)
Lung wheezing rales	3 (5.8%)
**Microbiological findings**	13 (25%)
**Positive bacterial cultures of Sputum**	11 (21.2%)
Spn	8 (15.4%)
Sa	2 (3.8%)
Pae	1 (1.9%)
**Positive bacterial cultures of BALF**	2 (3.8%)
Spn	1 (1.9%)
Sa	1 (1.9%)
**Comorbidities**	
Rhinitis	7 (13.5%)
Sinusitis	5 (9.6%)
Laryngomalacia	3 (5.8%)
Airway stenosis	1 (1.9%)
**Treatment**	
Antibiotic	
Amo	6 (11.5%)
Cep (oral)	15 (28.8%)
Cep (intravenous)	26 (50%)
Azi	5 (9.6%)
Duration	3d-2w
**Effects**	
Remission	20 (38.5%)
Improve	24 (46.2%)
No improvement	8 (15.3%)
**Complication**	0

Values are expressed as n (%). M: male; F: female. Spn: *Streptococcus pneumonia;* Sa*: Staphylococcus aureus;* Pae: *Pseudomonas aeruginosa*. Amo:Amoxicillin-clavulanate; Cep: cephalosporin antibiotics; Azi: Azithromycin.

### Clinical characteristics of patients with PBB

Nineteen patients had a duration of cough more than 4 weeks, 14 patients had 4–12 weeks, and more than 12 weeks in 19 patients. The longest duration was 28 weeks. All of the children presented wet cough, including 13 patients with purulent sputum, and 22 patients were coughing both day and night**.** Fifteen cases accompanied by wheezing, 11 patients accompanied by mild to moderate fever, and 19 patients had nasal symptoms, including nasal congestion, runny nose, and sneezing. Seven patients accompanied by rhinitis and 5 patients had sinusitis. Physical examination revealed that 30 patients had crackles, 3 patients accompanied by crackles and wheezes (Table 1).

### Laboratory characteristics of patients with PBB

Blood routine examination revealed that 14 cases had increased leucocyte and 9 cases with increased neutrophils. All cases had normal CRP, T cell subset, immune globulin, and NK cell. No patient showed a positive result when blood samples were collected for respiratory virus detection (respiratory syncytial virus, adenovirus, and parainfluenza virus type 1, 2, 3). A total of 14 patients underwent bronchoscopy, and all cases revealed mucosal congestion; 12 cases had thin secretions, 2 cases having purulent secretions. Laryngomalacia was identified in 3 cases, 1 case with tracheal stenosis. Positive bacterial cultures were confirmed in 25% of cases (13/52 cases). *Streptococcus pneumoniae* (Spn) was found in nine cases, *Staphylococcus aureus* (Sa) in three cases, and *Pseudomonas aeruginosa* (Pae) in one case. Among them, one case of Sa and one case of Spn were found in BALF. Forty-five children received a chest X-ray examination, 27 cases showed increased lung markings. The chest CT of five cases was normal (Table 1).

### Treatment and follow-up

All patients received antibiotics treatment for 3–7 days before our treatment, however, symptoms received no improvement or repeated after the withdrawal. In all enrolled patients, 6 patients were treated with oral amoxicillin-clavulanate (Amo) and 15 patients received oral cephalosporin antibiotics. Moreover, 26 patients received intravenous cephalosporin, and 5 children were administrated with Azithromycin (Azi) because they were allergic to penicillin and cephalosporins. All enrolled patients underwent outpatient follow-up every 2 weeks for a total of 4 weeks. Cough in the 24 hospitalized children was improved significantly when discharged from hospital after treatment for 3 to 7 days. Among the 28 outpatients, 20 cases received a resolution of cough after 2 weeks of treatment, while 8 cases still had a cough, including 5 cases with irregular medication and 3 cases accompanied by rhinitis (Table 1).

### Article review

Fourteen studies were identified (7 studies in China and 7 studies in the western countries, *N* = 660). We further analyzed the data to confirm whether the clinical feature is similar or not in the east and the west. All of the studies in China were retrospective designs, which showed a high proportion of male and most of them were younger than 3 years old. Fifty two patients performed antibiotic treatment before sputum culture and 178 cases (178/218, 81.7%) underwent airway bronchoscopy. The proportion of positive pathogen culture was 63.3% (138/218 cases), other cases were diagnosed as clinically-based-PBB. The detected bacteria were Spn (63/138 cases, 45.7%), *Haemophilus influenza* (Hi) (41/138 cases, 29.7%), *Moraxella catarrhalis* (Mcat) (20/138 cases, 14.5%), Sa (6/138 cases, 4.4%), Pae (3/138 cases, 2.2%), *Klebsiella pneumonia* (2 cases, 1.4%)*, Escherichia coli* (2 cases, 1.4%) *and Enterobacter aerogenes* (1 case, 0.7%). Furthermore, PBB was often accompanied by wheezing and was partially associated with sinusitis, airway stenosis, and tracheobronchomalacia. Most cases improved and some relapsed after treatment with Amo (Table 2).

**Table 2:** Clinical characteristics of PBB in China

Author	Li [6]	Li [7]	Chen [8]	Shi [9]	Gao [10]	Chi [11]	Shun [12]
**Year**	2018	2017	2016	2016	2016	2015	2014
**Number**	50	30	31	31	20	28	28
**M(F)**	36 (14)	18 (12)	18 (13)	17 (14)	–	26 (2)	17 (11)
**Age**	3.2y (5 m-14y)	14.5 m (7-49 m)	–	<3y	2 m-14y	8.5 m	6-75 m
**Course**	2 m	9.7w (5.7–7.1w)	2 m	4-11w	>4w	4.2w	>4w
**Antibiotic used**	–	20	11	–	–	–	21
**Bronchoscopy**	50	30	31	31	0	28	8
**Bacteria**							
Spn	14 (28%)	12 (40%)	6 (19.4%)	16 (51.6%)	2 (10%)	5 (17.9%)	8 (28.6%)
Hi	5 (10%)	10 (33.3%)	7 (22.6%)	3 (9.7%)	3 (15%)	3 (10.7%)	10 (35.7%)
Mcat	4 (8%)	7 (23.3%)	3 (9.7%)	–	–	–	6 (21.4%)
Sa	4 (8%)	–	2 (6.5%)	–	–	–	–
Kpn	–	–	–	2 (6.5%)	–	–	–
Eco	–	–	–	1 (3.2%)	–	1 (3.6%)	–
Pae	–	–	–	3 (9.7%)	–	–	–
Eae	–	–	–	–	–	1 (3.6%)	–
**Comorbidities**							
Wheeze	30 (60%)	22 (73.3%)	17 (54.8%)	25 (80.6%)	20 (100%)	21 (75%)	–
Rhinitis	–	–	1 (3.2%)				
Sinusitis	14 (28%)	16 (53.3%)	9 (29%)	–	–	–	–
Airway stenosis	6 (12%)	5 (6%)	5 (16.1%)	2 (6.4%)	–	–	–
Tracheobronchial malacia	5 (10%)	5 (6%)	4 (12.9%)	3 (9.6%)	–	11 (39.2%)	4 (14.2%)
**Treatment**							
Antibiotic	Amo /Cep /Azi	–	Amo	Amo /Cep /Azi/Car/Tei/Lin	Amo	Amo /Cep	Amo /Cep /Azi
Duration	2-4w		>2w	4-6w	2w	17.3 + 3.2d	2w
Follow up	1y	1y	2 m-2y	0.5y	–	2w	–
**Effects**							
Remission	33	–	23	–	–	–	28
Improve	15	20	8	31	20	28	–
Relapse	–	2	–	–	–	–	–
**Complication**	–	1*	–	–	–	–	–

Values are expressed as n (%). M: male; F: female. Spn: *Streptococcus pneumonia;* Hi: *Haemophilus influenza*; Mcat: *Moraxella catarrhalis*; Sa*: Staphylococcus aureus;* Kpn: *Klebsiella pneumonia;* Eco: *Escherichia coli*; Pae: *Pseudomonas aeruginosa*; Eae: *Enterobacter aerogenes*. Amo:Amoxicillin-clavulanate; Cep: cephalosporin antibiotics; Azi: Azithromycin; Car: Carbapenems; Tei: Teicoplanin; Lin: linezolid. * bronchiectasia.

Children with PBB in the western countries were also common in male, most patients were younger than 6 years old. Nine patients performed antibiotic treatment before culture and 380 cases (380/442, 86%) underwent airway bronchoscopy. Co-infection could be found in the 442 cases of PBB, some patients were caused by multiple pathogenic species and some cases were co-infected by bacteria and viruses. The main pathogens were: Hi (191/471 cases, 40.5%), Mcat (137/471 cases, 29.1%), Spn (119/471 cases, 25.3%), and Sa (24/471 cases, 5.1%). Most patients accompanied by wheezing and tracheobronchomalacia. Antibiotic treatment for 2 weeks revealed improvement in most cases, however, 68 relapsed cases (most of the patients with tracheobronchial malacia) needed to extend the course of antibiotic treatment (Table 3).

**Table 3:** Clinical characteristics of PBB in the western countries

Author	Pritchard MG [13]	Wurzel DF [14]	Narang R [15]	Chang AB [16]	Kompare M [17]	Donnelly D [18]	Marchant JM [1]
**Year**	2015	2014	2014	2012	2012	2007	2006
**Research**	R	P	R	P	R	R	P
**Number**	44	104	50	50	70	81	43
**M(F)**	–	72 (32)	–	36 (14)	50 (20)	40 (41)	–
**Age**	2.7y (1.5–4.0)	19 m (12-30 m)	2.9y (1.7–4.4)	4.5y	3 m	3.9y (5 m-14y)	2.6y
**Course**	11 m (9–14.7 m)	28w (6-57w)	11 m (8-14 m)	>4w	1-60 m 5 m	>1 m	>3w
**Antibiotic used**	–	–	–	–	–	9	–
**Bronchoscopy**	44	104	50	50	70	19	43
**Pathogen**							
Hi	27 (61.3%)	75 (72.1%)	25 (50%)	5 (10%)	39 (55%)	65 (80%)	20 (46.5%)
Mcat	22 (50%)	45 (43.3%)	14 (28%)	4 (8%)	41 (58.6%)	–	11 (25.5%)
Spn	10 (22.7%)	41 (39.4%)	8 (16%)	19 (38%)	26 (37.1)	30 (37%)	15 (34.9%)
Sa	8 (18.1%)	–	11 (22%)	4 (8%)	–	–	–
Adv	–	22 (21.1%)	–	–	–	–	2 (4.6%)
RSV	–	5 (4.8%)	–	–	–	–	–
PIV	–	–	–	–	–	–	1 (2.3%)
**Comorbidities**							
Wheeze	–	63 (60.5%)	–	30 (60%)	–	39 (48.1%)	–
Sinusitis	–	–	–	14 (28%)	–	–	–
Airway stenosis	–	–	–	6 (12%)	–	–	–
Tracheobronchial malacia	–	71 (68.2%)	–	5 (10%)	52 (74.2%)	–	–
**Treatment**							
Antibiotics	Amo /Cla	Amo	Amo /Cla	Amo /Cep /Azi	Amo	Amo	Amo
Duration	6-8w	2w	6w	2-4w	2-4w	4-6w	2w
**Follow-up**	1-4y	–	–	1y	–		1y
**Effects**			–				
Remission	–	–	–	–	–	52	–
Improve	33	104	–	50	61	–	43
Relapse	25	–	–	–	43	–	–
**Complication**	–	–	–	–	–	–	–

Values are expressed as n (%). R: retrospective research, P: prospective research. M: male; F: female. Hi: *Haemophilus influenza*; Mcat, *Moraxella catarrhalis*; Spn: *Streptococcus pneumonia;* Sa*: Staphylococcus aureus;* Adv:adenovirus; RSV:respiratory syncytial virus; PIV:parainfluenza virus. Amo:Amoxicillin-clavulanate; Cla: Clarithromycin; Cep: cephalosporin antibiotics; Azi: Azithromycin.

**Fig. 1:**
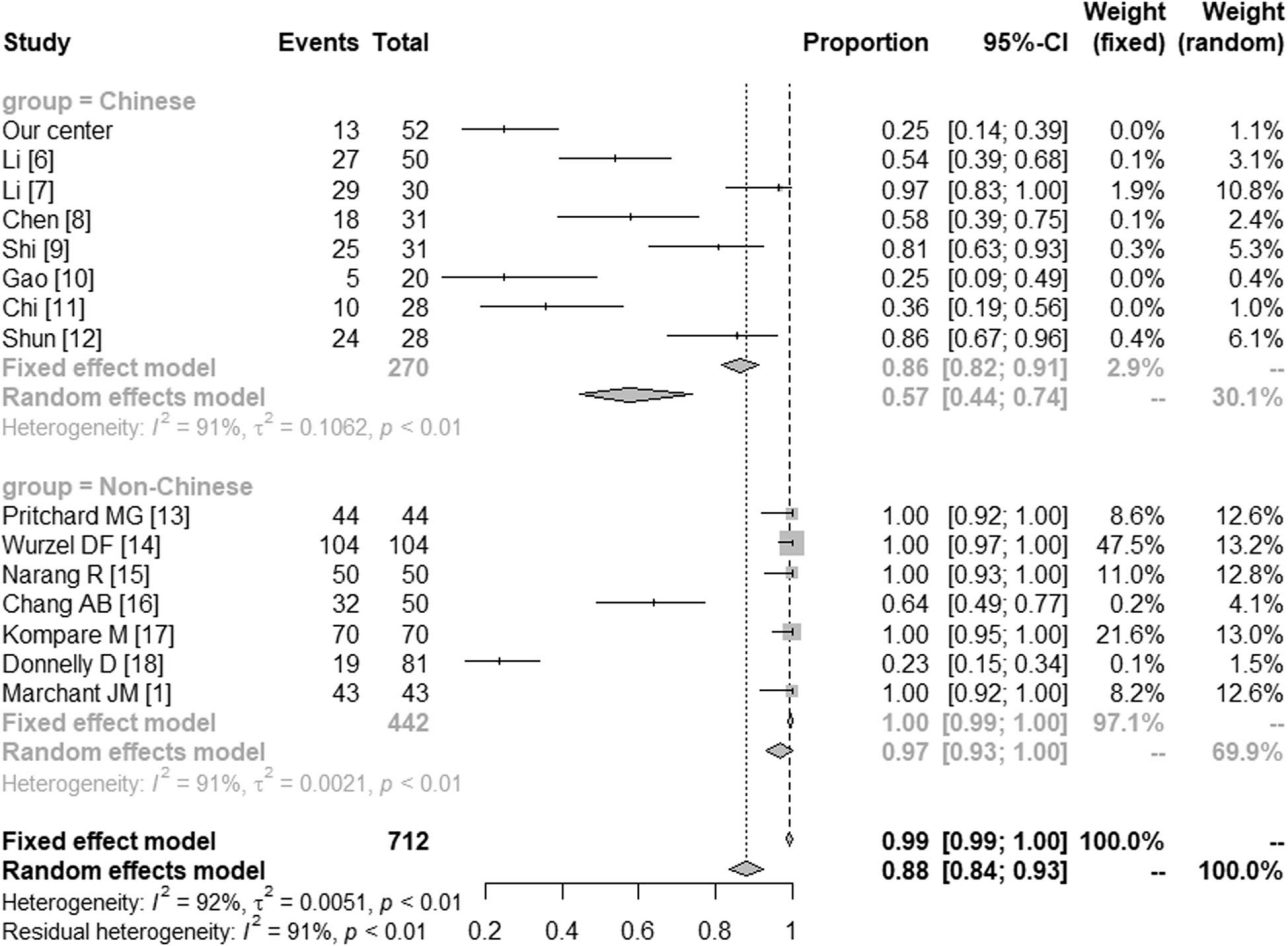
Microbiologically-based-PBB in China and the western countries. CI: Confidence Interval

**Fig. 2:**
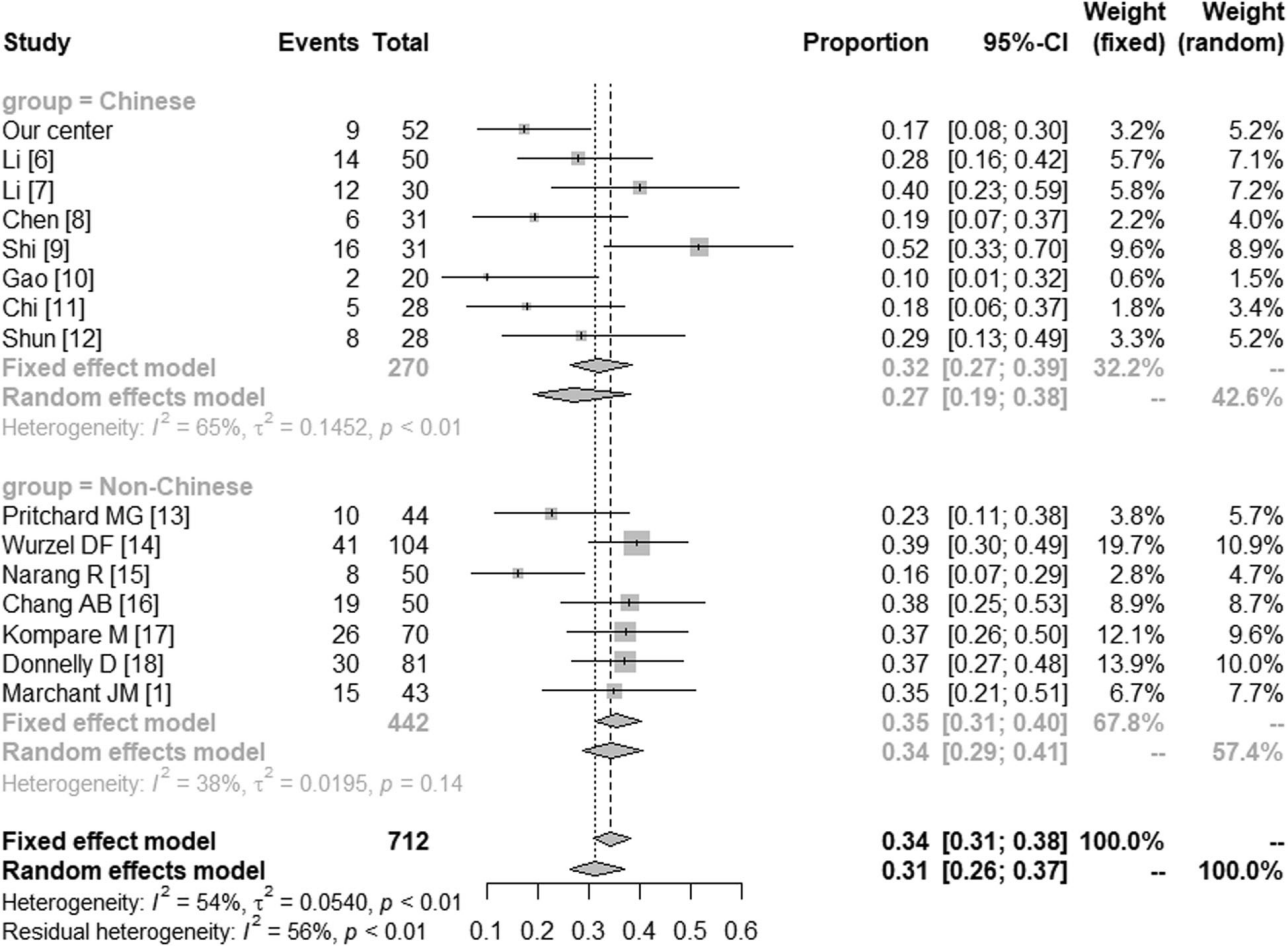
*Streptococcus pneumonia* in China and the western countries. CI: Confidence Interval

**Fig. 3:**
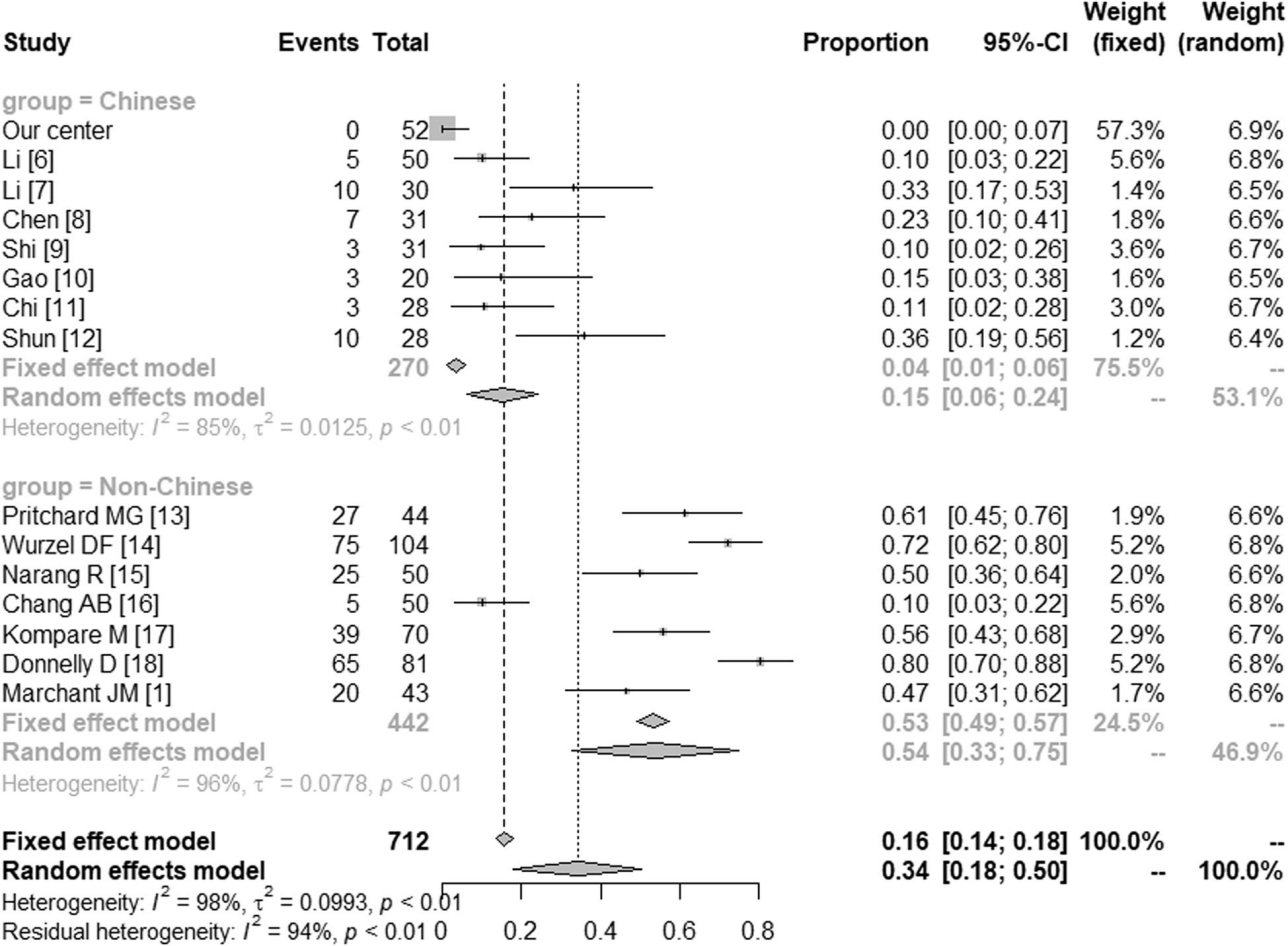
*Haemophilus influenza* in China and the western countries. CI: Confidence Interval

**Fig. 4:**
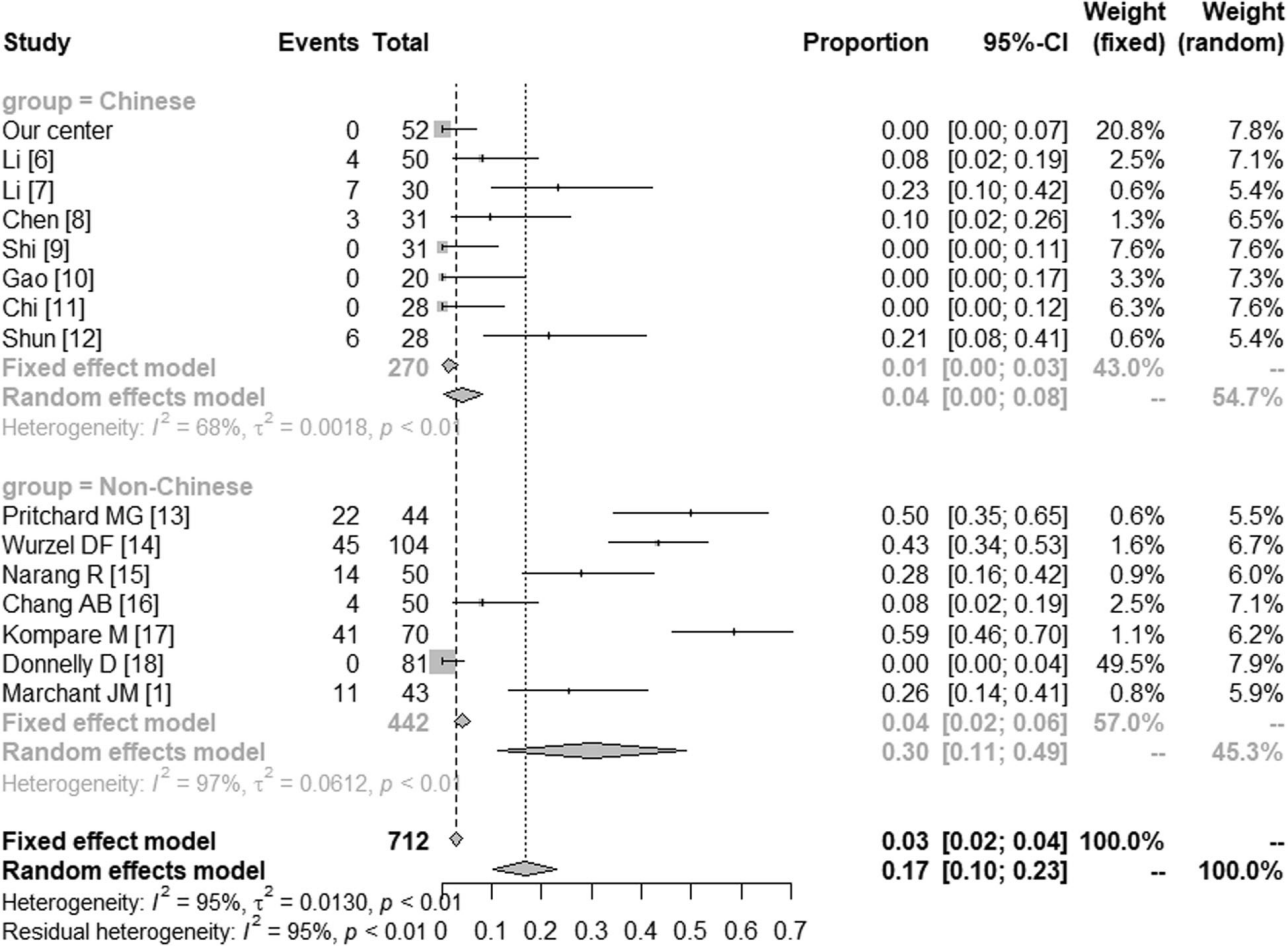
*Moraxella catarrhalis* in China and the western countries. CI: Confidence Interval

The microbiologically-based-PBB and the main pathogens in China and in the western countries were further compared by meta-analysis. The microbiologically-based-PBB in China (Proportion = 0.57 [0.44; 0.74]) and in the western countries (Proportion = 0.97 [0.93; 1.00]) were shown in Fig. 1. In Fig. 2, we have compared the *Streptococcus pneumonia* in Chinese (Proportion = 0.27 [0.19; 0.38]) and non-Chinese (Proportion = 0.34 [0.29; 0.41]). The *Haemophilus influenza* in China (Proportion = 0.15 [0.06; 0.24]) and in the western countries (Proportion = 0.54 [0.33; 0.75]) were shown in Fig. 3. And Fig. 4 illustrated the *Moraxella catarrhalis* in Chinese (Proportion = 0.04 [0.00; 0.08]) and non-Chinese cases (Proportion = 0.30 [0.11; 0.49]).

## Discussion

PBB is also known as persistent endobronchial infection and chronic bronchitis of childhood [4]. It was the most frequent etiologies in children with chronic cough [19], however, it has remained largely unrecognized [20]. To make an accurate diagnosis and a therapeutic approach to this common disease in children is needed [21]. This study analyzed the clinical characteristics of PBB in our center and in the published clinical studies, in order to deepen the understanding of PBB and provide a partial basis for the management of PBB.

The confirmed diagnosis of PBB depended on positive bacterial cultures of sputum or BALF [5]. Most of the cases were clinically diagnosed PBB and only 25% of patients were diagnosed as microbiologically-based-PBB among the 52 children enrolled in our study. Studies published in China showed that microbiologically-based-PBB was 63.3%. In other words, there were more than 30% of PBB cases were diagnosed as clinically-based-PBB. However, the number of microbiologically-based-PBB was higher in western countries than that in China. The lower rate of the positive pathogen may be related to the small number of cases who underwent bronchoscopy because it is impractical to conduct bronchoscopy for every child with chronic wet cough [5]. Young children usually couldn’t expectorate efficient sputum for culture and the effect of antibiotic therapy before enrollment may also contribute to the negative culture. A study reported that the culture results of bronchial aspirates were the same with BALF in some of the cases [22]. Therefore, bronchial aspirates may replace BALF in patients who couldn’t tolerate a lavage or expectorate sufficient sputum for reliable culture.

For the pathogen analysis, the most common pathogens in our center were Spn (17.3%) and Sa (5.8%), and the top five pathogens were Spn, Hi, Mcat, Sa, and Pae in the published Chinese research. The data from the western countries revealed that the main pathogenic bacteria were Hi, Mcat, Spn, and Sa. The top three pathogens were the same in the east and west, and Spn and Hi were the most frequent etiology in China and the western countries, respectively. This result may be caused by the inconsistent distribution of pathogenic bacteria and the choice of antibiotics in different countries and regions. Co-infection could be found in the identified studies, some patients were caused by multiple bacteria and several cases were co-infected by bacteria and viruses [1, 14]. Consistent with the findings, microbiota dysbiosis can be found in the BALF of patients with PBB [23] and the inflammation may not be driven by single bacteria [24]. Viruses also can be detected in BALF of patients with PBB, however, there was no evidence to prove that PBB was virus-induced [25].

Studies showed that PBB occurred mainly in younger than 3 years old males [26]. In China, the youngest patient was 2 months old (median age was 8.5 months) and the duration of cough varied from 4 weeks to 2 months [11]. The onset age of patients with PBB in other countries was 3 months old (average age was 6 years old) and the longest duration was 60 months [14, 16, 17]. Our result was consistent with previous studies, most patients were less than 6 years old males, and the longest duration was 28 weeks, suggesting infants and preschool children with a chronic wet cough should be alert to be PBB. Some patients with PBB presented with wheezing, and some may co-exist with asthma [5]. The prospective studies [14, 16] found that approximately 60% of children with PBB were accompanied by wheezing, wherera a study reported wheezing could be observed in all children with PBB [10]. Consistent with those findings, wheezing was the most common symptom in children with PBB in our study. PBB with chronic cough and wheezing was likely to misdiagnosis as asthma [13]. These children can be distinguished from asthma which was characterized by a dry cough and effective corticosteroid treatment [4, 27]. However, some children with unsatisfactory effects after antibiotic therapy should pay attention to the diagnosis of asthma because some PBB were finally diagnosed as asthma. In addition, the prolonged chronic wet cough of PBB may be associated with airway deformities which led to the dysfunction of cilia, mucus retention, and secondary infection. There were 3 cases of laryngomalacia and 1 case of airway stenosis in our data. Tracheobronchomalacia and airway stenosis was observed in patients with PBB in China. Wurzel [14] reported that 68.3% of PBB accompanied by tracheobronchomalacia (71/104 cases) and 74% (52/70 cases) of children were combined with airway malacia [17], suggesting that airway deformities were also common in patients with PBB in the western countries.

Amo was preferred for the treatment of PBB since it was sensitive to the common pathogens [5]. Studies showed that the improvement of cough symptoms required antibiotic treatment at least 10–14 days. Most children received a resolution of cough after amoxicillin-clavulanate treatments for 2–4 weeks, while cough relapsed again in some cases with tracheobronchial malacia [17]. Data in China found that most children treated with antibiotics received a resolution of cough, while certain patients with comorbidities (rhinitis, sinusitis) and poor compliance exhibited recurrent symptoms and unsatisfactory effects [7]. A retrospective study involving 81 children with PBB showed that cough relapsed in a large proportion of patients who received 2 weeks of antibiotic treatment and 13% of them needed a longer course of treatment [18]. The inflammation of PBB related to NLRP3/IL-1β [28] and biofilm formation which was considered to be a reason for a longer period of antibiotic treatment [4]. Gross et al [29] suggested the duration of initial antibiotic treatment was associated with recurrent PBB because 6 weeks of antibiotics treatment reduced the recurrent PBB than 2 weeks of treatment. Given cough was improved significantly in all patients who treated in hospital in our center, while 8 in 28 outpatients still had a cough. We proposed whether the patients’ compliance in the different settings of treatment will affect the resolution of the symptoms. Therefore, 2 weeks of Amo treatment was considered to be reasonable, however, further investigations (such as comorbidities, bronchoscopy, culture of BALF, and patients’ compliance) should be undertaken and the duration of treatment should be extended if symptoms persisted or relapsed [29, 30]. The vaccine may benefit to the management of PBB for it can decrease the respiratory symptoms and antibiotics course [31].

Studies suggested that PBB, chronic suppurative lung and bronchiectasis were in a dynamic development process. PBB was considered to be the early stage of chronic suppurative lung disease and it shared some similarities with early bronchiectasis [32]. PBB and bronchiectasis had similar gene expressions related to macrophage function and resolution of inflammation [33]. Although cause and effect were unproven, some repeated PBB may result in progress to bronchiectasis [4]. Li [7] reported a patient with PBB who developed to be bronchiectasis after a 1-year follow-up. A study revealed 13 children with PBB were diagnosed with bronchiectasis after 2 years follow-up in a prospective cohort study with 161 patients; Hi infection and recurrent PBB were the major risks for bronchiectasis [34]. Therefore, the possibility of bronchiectasis should be paid attention to patients with Hi infection and repeated PBB.

This study has the limitation that the sample size of our center was relatively small. The second limitation is the possibility of bias may exist because of the retrospective design. In addition, the follow-up was relatively short which may be difficult to reflect the outcome of PBB. Nevertheless, the combined analysis of the published data from the east and west limits this potential bias.

## Conclusions

In conclusion, PBB is common in male infants with chronic wet cough and may be accompanied by wheezing and airway deformities. Most cases are clinically diagnosed PBB in China and microbiologically-based-PBB is common in western countries. Co-infection could be found, and Spn, Hi, Mcat are the main pathogens. Spn and Hi were the most frequent etiology in China and western countries, respectively. Patients with comorbidities may need extended antibiotics treatment for more than 2 weeks.

## Data Availability

All data in this study are available from the corresponding author on reasonable request.

## Abbreviations

Adv: Adenovirus
Amo: Amoxicillin-clavulanate
Azi: Azithromycin
BALF: Bronchoalveolar lavage fluid
Car: Carbapenems
Cep: Cephalosporin antibiotics
CI: Confidence Interval
Cla: Clarithromycin
Eae: *Enterobacter aerogenes*
Eco: *Escherichia coli*
F: Female
Hi: *Haemophilus influenza*
Kpn: *Klebsiella pneumonia*
Lin: Linezolid
M: Male
Mcat: *Moraxella catarrhalis*
Pae: *Pseudomonas aeruginosa*
PBB: Protracted bacterial bronchitis
PIV: Parainfluenza virus
RSV: Respiratory syncytial virus
Sa: *Staphylococcus aureus*
Spn: *Streptococcus pneumonia*
Tei: Teicoplanin

## References

[1] Marchant JM, Masters IB, Taylor SM, Cox NC, Seymour GJ, Chang AB (2006). Evaluation and outcome of young children with chronic cough. Chest.

[2] Chang AB, Oppenheimer JJ, Weinberger MM, Rubin BK, Grant CC, Weir K (2017). Management of Children with Chronic wet Cough and Protracted Bacterial Bronchitis: CHEST guideline and expert panel report. Chest.

[3] Craven V, Everard ML (2013). Protracted bacterial bronchitis: reinventing an old disease. Arch Dis Child.

[4] Das S, Sockrider M (2018). Protracted bacterial bronchitis (PBB) in children. Am J Respir Crit Care Med.

[5] Chang AB, Upham JW, Masters IB, Redding GR, Gibson PG, Marchant JM (2016). Protracted bacterial bronchitis: the last decade and the road ahead. Pediatr Pulmonol.

[6] Changchang Li, Lin Dong, Yongqiang Xia. Case-control study for protracted bacterial bronchitis. J Wenzhou Med Univ. 2018;48:837–841. (published in Chinese).

[7] Yin Li, Xiaohong Xie , Luo Ren. Clinical characteristics and follow-up results of 30 cases of protracted bacterial bronchitis in children. J Appl Clin Pediatr. 2017;32:1231–1234. (published in Chinese).

[8] Jiehua Chen, Zhichuan Li, Hongling Ma, Wenjian Wang, Jiangqiang Xu, Yuejie Zhen. Clinical features and treatment of protracted bacterial bronchitis in children. J Clin Pediatr. 2016;34:575–579. (published in Chinese).

[9] Junran Shi, Jinrong Liu, Huimin Li, Wei Wang, Shunying Zhao. Analysis on clinical feature of 31 protracted bacterial brochitis in children. Chin J Pediatr. 2016;54:527–530. (published in Chinese).

[10] Yanmin Gao, Qing Chang, Rong Yu, Pan Zhang, Chenna Peng. Clinical feature of 20 protracted bacterial bronchitis in children. Chinese J Convalescent Med. 2016;25:875–876. (published in Chinese).

[11] Fanfan Chi, Yuqing Wang, Chuanli Hao, et al Analysis on clinical feature of 28 protracted bacterial brochitis in Children. Chin J Pediatr. 2015;53:784–787. (published in Chinese). 26758117

[12] Yin Shun. Analysis on clinical feature of 28 protracted bacterial brochitis in children. Shandong University, 2014. (published in Chinese).

[13] Pritchard MG, Lenney W, Gilchrist FJ (2015). Outcomes in children with protracted bacterial bronchitis confirmed by bronchoscopy. Arch Dis Child.

[14] Wurzel DF, Marchant JM, Yerkovich ST, Upham JW, Mackay IM, Masters IB (2014). Prospective characterization of protracted bacterial bronchitis in children. Chest.

[15] Narang R, Bakewell K, Peach J, Clayton S, Samuels M, Alexander J (2014). Bacterial distribution in the lungs of children with protracted bacterial bronchitis. PLoS One.

[16] Chang AB, Robertson CF, Van Asperen PP, Glasgow NJ, Mellis CM, Masters IB (2012). A multicenter study on chronic cough in children : burden and etiologies based on a standardized management pathway. Chest.

[17] Kompare M, Weinberger M (2012). Protracted bacterial bronchitis in young children: association with airway malacia. J Pediatr.

[18] Donnelly D, Critchlow A, Everard ML (2007). Outcomes in children treated for persistent bacterial bronchitis. Thorax.

[19] Ilarslan NEC, Gunay F, Haskologlu ZS, Bal SK, Tezcaner ZC, Kirsaclioglu CT (2019). Evaluation of children with chronic cough including obstructive sleep apnea: a single-center experience. Eur J Pediatr.

[20] Kantar A, Chang AB, Shields MD, Marchant JM, Grimwood K, Grigg J, et al. ERS statement on protracted bacterial bronchitis in children. Eur Respir J. 2017;50. 10.1183/13993003.02139-2016 28838975

[21] Korppi M (2019). Review shows paediatric protracted bacterial bronchitis needs an accurate diagnosis and strictly targeted extended antibiotics. Acta Paediatr.

[22] Verhulst S, Boel L, Van Hoorenbeeck K (2019). Protracted bacterial bronchitis: bronchial aspirate versus bronchoalveolar lavage findings: a single-Centre retrospective study. BMJ Paediatr Open.

[23] Bao Y, Li Y, Qiu C, Wang W, Yang Z, Huang L (2018). Bronchoalveolar lavage fluid microbiota dysbiosis in infants with protracted bacterial bronchitis. J Thorac Dis.

[24] Marsh RL, Smith-Vaughan HC, Chen ACH, Marchant JM, Yerkovich ST, Gibson PG (2019). Multiple respiratory microbiota profiles are associated with lower airway inflammation in children with protracted bacterial bronchitis. Chest.

[25] Wang Y, Hao C, Ji W, Lu Y, Wu M, Chen S (2019). Detecting respiratory viruses in children with protracted bacterial bronchitis. Respir Med.

[26] Wang Y, Hao C, Chi F, Yu X, Sun H, Huang L (2015). Clinical characteristics of protracted bacterial bronchitis in Chinese infants. Sci Rep.

[27] Bidiwala A, Krilov LR, Pirzada M, Patel SJ (2015). Pro-con debate: protracted bacterial bronchitis as a cause of chronic cough in children. Pediatr Ann.

[28] Chen AC, Tran HB, Xi Y, Yerkovich ST, Baines KJ, Pizzutto SJ, et al. Multiple inflammasomes may regulate the interleukin-1-driven inflammation in protracted bacterial bronchitis. ERJ Open Res. 2018;4. 10.1183/23120541.00130-2017 PMC5868518 29594175

[29] Gross-Hodge E, Carroll WD, Rainford N, Gamble C, Gilchrist FJ. Duration of initial antibiotic course is associated with recurrent relapse in protracted bacterial bronchitis. Arch Dis Child. 2019. 10.1136/archdischild-2019-317917 31624061

[30] Marchant J, Masters IB, Champion A, Petsky H, Chang AB (2012). Randomised controlled trial of amoxycillin clavulanate in children with chronic wet cough. Thorax.

[31] O'Grady KF, Chang AB, Cripps A, Mulholland EK, Smith-Vaughan H, Wood N (2018). The clinical, immunological and microbiological impact of the 10-valent pneumococcal-protein D conjugate vaccine in children with recurrent protracted bacterial bronchitis, chronic suppurative lung disease and bronchiectasis: a multi-Centre, double-blind, randomised controlled trial. Hum Vaccin Immunother.

[32] Chang AB, Marchant JM (2019). Protracted bacterial bronchitis is a precursor for bronchiectasis in children: myth or maxim?. Breathe (Sheff).

[33] Chen AC, Pena OM, Nel HJ, Yerkovich ST, Chang AB, Baines KJ (2018). Airway cells from protracted bacterial bronchitis and bronchiectasis share similar gene expression profiles. Pediatr Pulmonol.

[34] Wurzel DF, Marchant JM, Yerkovich ST, Upham JW, Petsky HL, Smith-Vaughan H (2016). Protracted bacterial bronchitis in children: natural history and risk factors for bronchiectasis. Chest.

